# Cardiac Pacing and Defibrillation in Children and Young Adults

**DOI:** 10.1016/s0972-6292(16)30584-8

**Published:** 2013-01-01

**Authors:** Harinder R Singh, Anjan S Batra, Seshadri Balaji

**Affiliations:** 1 The Carman and Ann Adams Department of Pediatrics, Children's Hospital of Michigan; 2 Department of Pediatrics, Children's Hospital of Orange County; 3 Department of Pediatrics, Oregon Health and Science University

**Keywords:** Pacemakers, pacing, ICDs, pediatrics, congenital heart defects

## Abstract

The population of children and young adults requiring a cardiac pacing device has been consistently increasing. The current generation of devices are small with a longer battery life, programming capabilities that can cater to the demands of the young patients and ability to treat brady and tachyarrhythmias as well as heart failure. This has increased the scope and clinical indications of using these devices. As patients with congenital heart disease (CHD) comprise majority of these patients requiring devices, the knowledge of indications, pacing leads and devices, anatomical variations and the technical skills required are different than that required in the adult population. In this review we attempt to discuss these specific points in detail to improve the understanding of cardiac pacing in children and young adults.

## Introduction

Pediatric pacing has progressed substantially since the first implant in a 14 yr old with myocarditis in 1962 .[1] Current pacemakers have a much smaller size, longer battery life, multiple pacing and sensing modalities, and therapeutic capabilities in the form of detecting and treating tachy-arrhythmias as well improving the contractility of a failing heart. Hence there is an increasing demand for pediatric pacing devices due to increase in clinical indications, technological advances and innovative techniques. However based on the 2010 Health Care Cost and Utilization Project (HCUP) database, only 0.6% of all the implanted cardiac devices have been in the pediatric population. The number of pediatric patients receiving pacemaker implantation has been stable over the past decade; however there has been a 4-fold rise in the number of patients receiving defibrillators and biventricular devices.[2]

## Indications

### a. Permanent Pacemakers

The most common indications for permanent pacemaker implantation in children, adolescents, and patients with congenital heart disease are:

1) Symptomatic sinus bradycardia related to sinus node dysfunction, associated with poor cardiac output or to prevent episodes of recurrent atrial tachycardias.

2) Advanced second- or third-degree AV block, either congenital or postsurgical, when associated with low cardiac output, ventricular dysfunction, complex ventricular ectopy, syncope or potential of recovery is minimal, especially after cardiac surgery.[3]

Important considerations in children and young adults are 1) an increasing number of young patients are long-term survivors of complex surgical procedures for congenital heart defects that result in palliation rather than correction of circulatory physiology. The residua of impaired ventricular function and abnormal physiology may result in symptoms due to sinus bradycardia or loss of AV synchrony at heart rates that do not produce symptoms in individuals with normal cardiovascular physiology. Hence, the indications for pacemaker implantation in these patients need to be based on the correlation of symptoms with relative bradycardia rather than absolute heart rate criteria. 2) The clinical significance of bradycardia is age dependent; e.g. a heart rate of 45 bpm may be a normal finding in an adolescent, the same rate in a newborn or infant indicates profound bradycardia. 3) Significant technical challenges may complicate device and transvenous lead implantation in very small patients or those with abnormalities of venous or intracardiac anatomy. 4) As there are no randomized clinical trials of cardiac pacing in pediatric or congenital heart disease patients, the level of evidence for most recommendations is consensus based.

### b. Implantable Cardioverter-Defibrillators (ICDs)

ICDs are recommended for patients who have survived an episode of cardiac arrest, patients with poor cardiac function with evidence of moderate to severe heart failure, patients with inducible ventricular dysrhythmia in a setting of symptomatic CHD and in patients with genetic cardiomyopathy.

Sudden cardiac death (SCD) in childhood and adolescence is associated with congenital heart disease, cardiomyopathies, and genetic arrhythmia syndromes. There is paucity of clinical experience and data regarding ICD implantation for primary prevention of SCD in young patients and therefore recommendations are based on extrapolation of data from adult studies. Unexpected sudden death is reported in 1.2% to 3.0% of patients per decade after surgical treatment of tetralogy of Fallot, with risk factors including ventricular dysfunction, QRS duration, and atrial and ventricular arrhythmias.[4] A significantly greater risk of SCD has been identified for patients with transposition of the great arteries or aortic stenosis, with most cases presumed to be due to a malignant ventricular arrhythmia associated with ischemia, ventricular dysfunction, or a rapid ventricular response to atrial flutter or fibrillation.[5] The lack of prospective and randomized clinical trials precludes exact recommendations regarding risk stratification and indications for ICD implantation for primary prevention of SCD in patients with postoperative congenital heart disease and ventricular dysfunction. ICDs may also be considered as a bridge to orthotopic heart transplantation in pediatric patients, particularly given the longer times to donor procurement in younger patients.[6]

### c. Biventricular pacing (Cardiac Resynchronization Therapy, CRT)

There are no randomized multicenter studies regarding use of CRT in pediatrics and young adults. The limited worldwide pediatric experiences has shown that CRT is useful in select younger patients with clinical improvements comparable to adult patients and, in some instances, can delay or remove the need for heart transplant.[7]. The current guidelines for adults suggests biventricular pacing for patients with wide QRS rhythm of left bundle branch block morphology with ejection fraction ≤ 35% and in functional heart failure class 2-3 despite medical management.[8] These are not easily extrapolated to the pediatric population. The incidence of ischemic heart disease is very low in pediatric patients. Younger patients typically require pacing therapy for bradycardia associated with congenital heart block (often with normal ventricular contractility) or progressive damage to the atrioventricular conduction system following surgical repair of various structural congenital heart defects.

## Technical aspects of device implantation

The implantation of devices in children and young adults can be challenging especially in view of anatomical variations due to congenital defects and surgical procedures to repair the heart defects.

## Anatomical considerations

**Figure 1:**
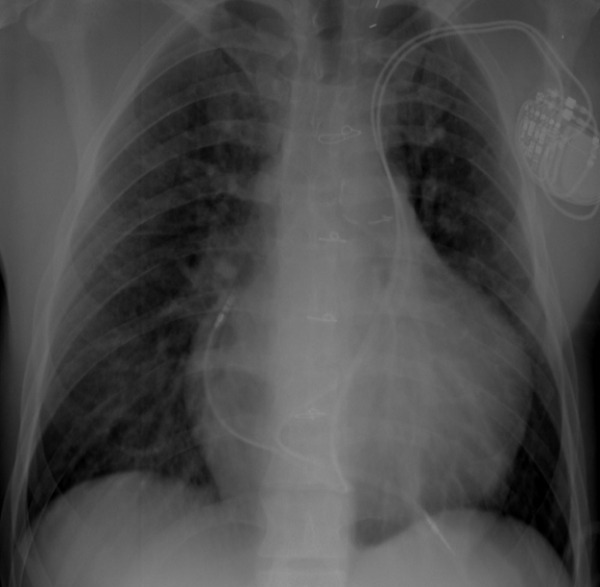
Dual chamber pacemaker lead implantation in a patient with left SVC without a bridging vein.

It is important to understand the anatomy and have a thorough knowledge of any underlying heart defects, presence of intracardiac shunts and type(s) of surgical procedure(s) if any performed in the past. Venography may help define presence or absence of left superior vena cava, any obstruction, or anomalies as well as patency of the vasculature if previous leads are present. Patients with d-transposition of the great arteries (d-TGA) with atrial switch operation have surgical baffles connecting the superior vena cava (SVC) and inferior vena cava to the pulmonary (left) ventricle. If placement of a lead is anticipated and there is presence of narrowing across the superior baffle, it is useful to consider stent angioplasty of the SVC baffle prior to lead implantation. Patients with previous cardiac surgery may have their right atrial appendage amputated at the time of canulation. In patients with Fontan palliation for single ventricular physiology, an atrial lead may be implanted transvenously in patients with atrio-pulmonary connection or lateral tunnel palliation (but not the extracardiac conduit), keeping in mind that they have a passive venous flow circulation. The presence of left SVC without a bridging vein can make the implantation technically challenging albeit possible (Figure 1). Future growth of the patient must be taken into account during lead implantation.

## Route of lead implantation

The pacing and defibrillator leads can be implanted via the transvenous (endocardial) or surgical (epicardial) route. The choice of route is dependent upon the size of the patient, anatomy and surgical procedures performed. The primary risk factor for obstruction after pacemaker lead implantation in children was found to be related to the size of the lead as compared to the body surface area at implantation. A ratio > 6.6 mm2/m2 was found to best predict venous obstruction, with a sensitivity of 90% and specificity of 84%. This data can be used to aid the physician in selection of a single or dual chamber lead system appropriate for the patient's size, thus decreasing the risk of venous obstruction, and hence preserving venous access.[9] Patient age, body size and lead characteristics at implant do not appear to predict occlusion in patients aged over 3 years.[10] For patients less than 10-15 kilograms, intracardiac shunt lesions, prosthetic tricuspid valve and circumstances where the anatomy or surgical palliation precludes access via the transvenous route, epicardial implantation is the route of choice. Epicardial lead implantation requires sternotomy or thoracotomy or subxiphoid approach, and is associated with higher chronic stimulation threshold, higher lead failures and fractures and early depletion of battery life.[11-13] However it preserves the venous access for future use. There have been case reports and small series of patients less than 10 kg who have successfully undergone transvenous lead implantation.[14-16] Endocardial lead placement offers the advantages of avoidance of thoracotomy, lower pacing thresholds, and a lower incidence of lead fractures. However its disadvantages include a greater risk of lead dislodgment, venous occlusion, embolic vascular events, and endocarditis.[17]

## Programming of the device

Children have faster resting heart rates than adults and higher peak heart rates - it is not unusual for infants to have resting heart rates between 120 and 150 beats/min, and it is easy for children of all ages to attain sinus rates in excess of 200 beats/min during active play. Many pacemakers cannot pace at or track sinus rates beyond 180 beats/min and rates with defibrillators are even lower. These limits to the maximum tracking rate can result in a substantial decrease in exercise performance, peak oxygen consumption and anaerobic threshold.[18] In addition, higher heart rates result in increased battery utilization that can significantly impact the longevity of the pulse generators.

## Use of single chamber vs. dual chamber ICD

Younger patients have higher sinus rates during exertion and increased frequency of supraventricular tachycardias especially in patients with congenital heart defects. It has been reported that 30% of patients with ICDs and congenital heart disease will develop supraventricular tachyarrhythmias during follow-up.[19,20] Meta-analysis of data from patients with ICDs reveals evidence that in those with dual-chamber ICD's, arrhythmia discrimination shows improved detection specificity without jeopardizing sensitivity; however the proportion of patients with inappropriate therapy was still approximately 20%, despite sophisticated arrhythmia discrimination.[21] There is no evidence that empirical use of any dual-chamber pacing approach improves mortality, quality of life, or reduces heart failure, ventricular tachyarrhythmia, or atrial arrhythmia. Moreover, the pulse generator longevity is about one-third less in dual- versus single-chamber ICDs.[22] In the case of obligatory pacing for symptomatic sinus node dysfunction, a dual-chamber strategy for minimal pacing at all chamber levels is recommended.[23]

## Technique of device implantation

The handedness of the patient is determined as the device is preferably implanted on the non-dominant side. The procedure is usually performed under general anesthesia. Antibiotic coverage is provided during and immediately after the procedure.[24] Based on the size of the patient and the device as well as cosmetics, the site of implantation is chosen. Subcutaneous pocket or submuscular pocket is created and rinsed with antibiotic solution. There is no difference in the pacing, sensing thresholds or defibrillation thresholds for ICDs in either the subcutaneous or submuscular implantation.[25] Some prefer the submuscular implantation in extremely thin individuals with minimal fat tissue to prevent device erosion and in patients with or at risk of Twiddler's syndrome. The vein is accessed with modified Seldinger technique or a venous cutdown. The number of leads decides the number of access sites in the veins. We attempt to access the axillary vein to avoid the complication of subclavian crush at the site of the ligament, reduce the risk of pneumothorax or hemothorax and less cumbersome extraction if necessary. The axillary vein is accessed by creating a roadmap by either placing a pacing catheter or by performing a venogram in the innominate vein or the cubital vein from a peripheral venous line. The ventricular leads are usually implanted first. The RV septum is usually targeted with manually shaping the stylets and the positioning confirmed on a biplane fluoroscope. The RV low septum is targeted in most of the patients with a routine curve to the stylet that lets it across the tricuspid valve followed by a posterior smaller curve near the tip of the stylet to obtain a septal position. If implanting a LV lead, the coronary sinus is accessed with special sheaths, an angiogram performed to delineate the anatomy and choose the target vein. Once the target vein is identified, the LV lead is implanted over a guide wire. If a dual chamber pacemaker is planned, an atrial lead is implanted next. The site of the Bachmann's bundle is preferred as it is associated with lower far field R wave sensing, atrial synchronization and prevention of atrial arrhythmias.[26-28] The Bachmann's bundle is located in the posterior high right atrial septum near the superior vena cava. The site is easily accessible using long sheaths and manually shaped stylets that have a smaller curve than the routine J-shaped stylet for positioning the lead in the right atrial appendage.[29] Pacing and sensing thresholds are determined. Pacing from each lead at 10V is performed to determine any potential phrenic nerve stimulation. The leads are secured with stay sutures in the musculature and around the pacing lead sleeves and attached to the generator. If placing an ICD, the defibrillation threshold (DFT) is obtained by the upper limit of vulnerability (ULV) testing or the binary search method. The upper limit of vulnerability (ULV) is the weakest shock strength at or above which VF is not induced when the shock is delivered at any time during the vulnerable period, which is the portion of the cardiac cycle during which shocks induce VF. ULV testing can be applied at ICD implant to confirm a clinically adequate defibrillation safety margin without inducing VF in 75%-95% of ICD recipients.[30] The binary search algorithm uses step-wise successive shock energies depending on the success of the previous shock. The lowest energy that successfully terminates the ventricular tachycardia is termed as the DFT.[31] The generator is secured and the incision is closed in multiple layers. The ipsilateral arm is immobilized in a sling for a period of 1-2 weeks to prevent lead dislodgement. The incision is kept dry for 7-10 days.

**Figure 2:**
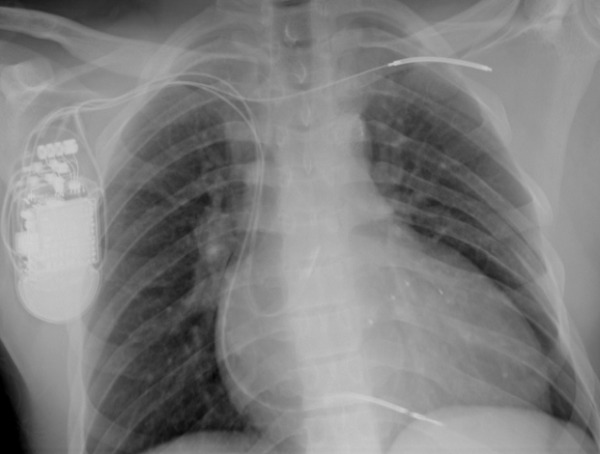
Use of an ICD coil in the left axillary vein in a patient with right sided generator implant to lower the DFTs. The atrial lead is in the posterior high right atrial septum near the Bachmann's bundle.

Based on variations in anatomy, the lead implantation technique may have to be revised. In patients with high DFTs at the time of ICD implantation, additional coils or subcutaneous array are implanted.[32] The placement of additional coils can be in the coronary sinus, azygous vein, or the left innominate or axillary vein (Figure 2). Implanting the device in the left axillary region has also been postulated to reduce the DFT.[33] Use of medications like Sotalol has been reported to lower the DFT .[34,35] In some group of patients a 'hybrid' approach to lead implantation is performed. If biventricular pacing is contemplated in patients with d-TGA with atrial switch palliation, a mini-sternotomy or thoracotomy is used to implant the systemic (RV) ventricular epicardial lead that is tunneled to the pocket where the generator with the transvenous leads is placed. In very small patients, ICD is implanted using a pericardial patch or a coil in the pericardial space with a bipolar sensing lead on the ventricle and implantation of the device in the abdomen.[36]

## Follow-up

The patient and the device are assessed prior to discharge, in 1- 2 weeks for incision check, at 2- 3 months to assess chronic pacing thresholds and cardiac function (because of the risk of pacing-induced cardiac dysfunction), and then 6 months to yearly. Patients are advised to transmit data using the remote monitoring services on a 3 monthly basis or if any change in clinical status occurs. The remote monitoring is intensified to a monthly basis in pacemaker dependent patients and in patients nearing end of battery life. Any patient experiencing an ICD discharge is recommended to follow up in the closest emergency room to evaluate the appropriateness of the discharge, need for in-patient admission, or pharmacological intervention. Chest radiographs have been advised on a yearly basis in small children to recognize any growth related lead damage. Echocardiograms are performed on an annual basis to evaluate valvular and cardiac function. Stress test is recommended to assess exercise tolerance, maximal heart rates achievable, assess the rate response settings, and exercise related arrhythmias for fine tuning of the device for allowing maximal functionality in children and young adults.

## Complications

Device implantation data using the Kids' Inpatient database from 1997-2006 revealed specific complication rates for all device types were pneumothorax 2.2%, hematoma 3.3%, endocarditis/ pericarditis 1.1%, surgical infection 2.4% and death 1.7%. Biventricular pacemakers have the highest percentage of acute complications (42.3%) whereas pacemakers (17.3%) and defibrillators (16.8%) were lower. Pacemakers had higher patient-related complications (11.2%) in comparison to ICDs and biventricular pacemakers and ICDs had higher device-related complications (11.5%) in comparison to the pacemakers and biventricular pacemakers.[2]

## Techniques of implantation to aid extraction

With the technological advances making more devices compatible for younger patients and the increasing population of adult congenital heart defect patients requiring device implantation, the need for revision or extraction will continue to increase. Considering lead implantation techniques and hardware that lend them to easier extractions would be helpful. Older lead age, a lead in the ventricular position, and polyurethane lead insulation were found to be independent predictors of the decreased likelihood of a simple extraction.[37] Long implantation time, lack of operator experience, ICD lead type and female gender are possible risk factors for life-threatening complications.[38] Implantation durations of less than 3 years had a success rate of 100% whereas it was only 65.5% in those that were older than 3 years, most probably due to robust fibrosis in the young patient population.[39] Medial subclavian vein approaches are discouraged due to the risk of crush requiring subsequent and likely difficult extraction. In the dual coil leads, the SVC coil stimulates more aggressive fibrosis with high risk for vascular tear at time of extraction.[40] Use of leads that are appropriately sized for the patient will reduce the amount of extra lead left in the pocket that may need to be dissected. Leads that are constructed well so as not to fall apart easily, and leads that are isodiametric with active fixation, are likely to be more easily and completely removed .[41] If passive fixation leads are to be used, shorter tine length will make extraction easier. As the IS-4 standard becomes widely available for ICD leads, this will eliminate the "yoke" on these leads, making dissection easier as well. The use of ICD leads that use coils backfilled with medical adhesive, or that are covered with Gortex™ markedly reduces the tissue in-growth and facilitates easier and safer extraction.[41,42]

## Conclusion

The utility of cardiac devices in the pediatric population is increasing due to technological advances as well as improved survival of patients with congenital heart defects. Symptomatic bradyarrhythmias, risk of sudden death, heart failure are the broad indications for implantation of a cardiac device. The selection of device and leads as well as the technique of implantation are based on the patient size and anatomy. Careful selection of the device, leads and technique can help reduce complications associated with the implantation as well as aid in extraction of the devices in the long term.
